# The Association between Cognitive Impairment and Diabetic Foot Care: Role of Neuropathy and Glycated Hemoglobin

**DOI:** 10.3390/pathophysiology27010003

**Published:** 2020-11-25

**Authors:** Lorenzo Brognara, Iacopo Volta, Vito Michele Cassano, Emmanuel Navarro-Flores, Omar Cauli

**Affiliations:** 1 Department of Biomedical and Neuromotor Sciences, University of Bologna, 40123 Bologna, Italy; lorenzo.brognara2@unibo.it (L.B.); iacopo.volta2@studio.unibo.it (I.V.); 2 Azienda Sanitaria Locale Toscana Nord-Ovest, Unitá Operativa Professioni della Riabilitazione, Cittadella della Salute, 55100 Lucca, Italy; vitomichele.cassano@uslnordovest.toscana.it; 3 Department of Nursing, Faculty of Nursing and Podiatry, University of Valencia, 46010 Valencia, Spain; emmanuel.navarro@uv.es; 4 Frailty Research Organized Group (FROG), University of Valencia, 46010 Valencia, Spain; 5 Chair for Healthy, Active and Participative Aging, University of Valencia, 46010 Valencia, Spain

**Keywords:** neuropathy, diabetes, skin diseases, diabetic foot complication, executive function, cognition, HbA1c

## Abstract

Diabetes mellitus is associated with impairment in cognitive functions which can complicate adherence to self-care behaviors. We evaluated the incidence of cognitive impairment in patients with diabetes mellitus to determine the strength of the association between diabetic foot (a complication that occurs in about 10% of diabetic patients), adherence to the clinician’s recommendations, glycemic control, and cognitive function. A prospective study was carried out in a probabilistic sample of older patients with diabetic foot living in three nursing homes. Cognitive functions were evaluated by the MMSE (Mini-Mental State Examination), the Trail Making test (TMT), and the Michigan neuropathy screening instrument (MNSI). There were no significant associations between cognitive function and neuropathy or foot alterations, although glycated hemoglobin (HB1Ac > 7%) significantly (*p* < 0.05) associated with MMSE and adherence to treatment in the 1 month follow-up visit. Receiver operating characteristic curve analysis showed that both HB1Ac and the MNSI score significantly (*p* < 0.05) discriminate subsequent adherence to treatment for foot complication, with a sensitivity of 80.0–73.3% and specificity 70.6–64.7%, respectively. Proper control of foot complications in diabetic patients involves appropriate glycemic control and less severe neuropathy, and seems to be unrelated to cognitive dysfunction, and warrants further studies in order to tailor appropriate treatments to central and peripheral nervous system disorders. Poor glycemic control (Hb1Ac level > 7%) and a neuropathy score of 5.5 in the MNSI are the best-cut off points to discriminate poor adherence to the clinician’s recommendations for self-care behaviors in people with diabetic foot complication. In this study, we observed that foot disorders were associated with impaired global cognitive function in elderly patients (aged ≥ 65). Podiatrists and physicians should consider cognitive dysfunction as an important chronic complication in the management of diabetic foot.

## 1. Introduction

Diabetic foot complication and management are a global public health problem, which affects 422 million people worldwide, is associated with high levels of morbidity and mortality, and occurs in around 10% of diabetic patients through their life. The major adverse outcome of foot ulceration is amputation, with a prevalence of 1.6% in the age range 18–44 years, 3.4% among those aged 45–64 years, and 3.6% in patients older than 65 years. It is estimated that 40% of patients have a recurrence within 1 year after the ulcer healing, almost 60% within 3 years, and 65% within 5 years. Mortality after diabetes-related amputation exceeds 70% at 5 years for all patients [1,2,3,4,5]. Various interventions for the prevention of foot ulcers have been studied, but adherence to treatment has now been confirmed as playing an important role in the clinical outcome [6,7]. Natovich et al. [8] have recently shown that those patients with diabetic foot adhered to physical activity to a significantly lesser extent than patients without diabetic foot. Patients who follow the recommendations have significantly better outcomes than those who do not [8,9]. The problem of nonadherence should guide practitioners when identifying patients who are nonadherent or anticipated to be nonadherent to clinical recommendations. This evidence attests to the fact that analysis of the factors affecting adherence to treatment among patients with diabetic foot complications are necessary to implement a proper educational intervention.

Recent studies have shown that adults with type 2 diabetes have evidence of slowing in brain activity on recordings of sensory-evoked potentials, and cerebral atrophy, white matter lesions and infarctions are frequently reported, and correlate with the presence of microvascular and macrovascular complications [10,11,12]. The literature contains a growing number of papers reporting on neurocognitive functioning in individuals with diabetes, and the publications in this field are constantly improving. A meta-analysis to determine the alterations in six cognitive domains in individuals with type 2 diabetes found a reduction in motor function, executive function, processing speed, verbal memory, and visual memory, but a preserved attention/concentration function in diabetic patients compared to nondiabetic individuals [13].

This neural slowing affects executive functions such as planning, attention, and problem solving [14,15,16,17,18]. In type 1 diabetes, magnetic encephalography detected abnormalities in functional magnetic fields and in the neural connectivity of the brain, regardless of the microvascular disease status [19,20]. Ryan et al. report that 24% of adolescents with early-onset diabetes (diagnosed before age 6 years) showed clinically significant impairments in a wide range of cognitive domains, compared with only 6% of the later-onset patients and 6% of people without diabetes [21,22].

The implementation of routine cognitive screening in individuals with diabetes-related foot ulcers has been proposed in recent years, and the need to detect and prevent cognitive impairment among patients with diabetic foot has also been emphasized [23]. Recent findings by Corbett et al. observed in patients hospitalized for diabetic foot ulcers showed that a reduced cognitive ability measured by the Montreal Cognitive Assessment was associated with a more limited understanding of peripheral neuropathy and foot ulcers, several misperceptions regarding peripheral neuropathy, less accurate attributions of blame to self or practitioners, and more accurate attributions of control of ulcer management to practitioners [24]. Practitioners should evaluate some items when educating the person at-risk for foot ulceration, such as determining whether the person is able to perform daily foot care management. Gegg et al. in a large prospective cohort study, showed that diabetes is associated with lower levels of cognitive function and greater cognitive decline among older women [25]. In addition, a longitudinal cohort study showed diabetes may be associated with an increased risk of developing Alzheimer’s disease and impaired cognitive function over time [26,27]. Dermatological abnormalities, skin manifestation, and cutaneous involvement are common, and affect 30% of people with diabetes mellitus [28,29]. A high incidence of cutaneous involvement may be used as an indicator of patients with cognitive and/or psychosocial impairment, and can lead to poorer self-care in several conditions [30,31,32].

Many dermatological problems are caused by hyperglycemia, and the pathogenesis is also caused by neuropathy, ischemia, and infection [3], but there are many factors that contribute to the development of skin diseases in older adults with diabetes mellitus, and some of these, such as cognitive function, have not been investigated in previous studies.

We hypothesize that the evaluation of cognitive impairment may be helpful in understanding the reasons for nonadherence among older institutionalized individuals, and help clinicians in this endeavor. In fact, a decline in mental efficiency might be sufficient to disrupt performance in diabetes self-management [9]. The aim of our study was to analyze the relationship between cognitive function, neuropathy, and diabetic foot complications, and to determine whether the level of cognitive function was associated with adherence to the clinician’s recommendations for self-care education. We evaluated the most common diabetic foot complication using the Michigan Neuropathy Screening Instrument (MNSII) that includes two assessments: a 15-item questionnaire, and a physical assessment and examination of the lower extremity that combines an evaluation of the appearance of feet ulceration and symmetrical peripheral neuropathy (ankle reflex, vibration perception in the big toe and monofilament). We used the Trail Making Test (TMT) and Mini-Mental State Examination (MMSE) to evaluate the cognitive functions. The MMSE is a well-known test for screening for cognitive impairment in older individuals in primary healthcare, and is widely used in screening for cognitive impairment in the diabetic population [33]. In order to detect cognitive alterations related to prefrontal cortex function, we also used the TMT (A and B), a neuropsychological test that evaluates psychomotor processing speed, executive function domain including divided attention and flexibility, which are functions mainly related to the prefrontal cortex [34,35]. We also investigated the clinical predictors for foot diseases and adherence to the clinician’s treatment recommendations during the follow-up (after one month) visit among patients with diabetes with cognitive impairment.

## 2. Materials and Methods

### 2.1. Sample Characteristics

A cross-sectional study was performed in individuals with diabetes and foot alterations (nursing home residents in Valencia, Spain). We screened and examined a total of 47 diabetic out-patients, and recorded demographic characteristics, HbA1c levels, and presence of foot complications. Patients affected by type 1 and type 2 diabetes with disease duration of more than 1 years, aged under 90 years, and treated with insulin and oral hypoglycemic agents, respectively, were enrolled in the study. The study protocol was approved by the Human Research Ethics Committee of the University of Valencia (Reference: H38417528). The study was performed between November 2019 and April 2020. Inclusion criteria were (1) proven clinical diagnosis of diabetes mellitus, (2) adult patients (aged 18 years and over). Exclusion criteria were (1) impaired vision (e.g., presence of retinopathy); (2) presence of hypoacusia limiting the ability to understand the questions in the instruments; (3) a severe peripheral vascular disease (ankle-brachial index < 0.90) as this has been demonstrated to be associated with cognitive impairment and dementia [36,37,38,39]; (4) a history of significant central nervous system impairment (e.g., previous cerebral stroke, head injury with loss of consciousness, Alzheimer’s disease, or other type of dementias) or severe psychiatric illness (e.g., schizophrenia or bipolar disorder) [40,41]. The sample size was calculated based on the number of diabetic patients with foot alterations attending podiatric consulting in three nursing homes, and considering a drop-out rate of 20% in the follow-up visit based on nursing home records. Using paired measurements (repeated in one group) (Proportions) and accepting an alpha risk of 0.05 and a beta risk of 0.2 in a two-sided test, 46 subjects were required to recognize a difference consisting in an initial proportion of 0.9 and a final proportion of 0.6 as statistically significant.

### 2.2. Evaluation of Cognitive Function

The MMSE scale is a widely used scale for assessing cognitive function and screening for dementia. It can be administered by clinicians or researchers with minimal training, takes around 10 min, and assesses cognitive function in the areas of orientation, memory, attention and calculation, language, and visual construction. Patients score between 0 and 30 points (higher MMSE scores mean better cognitive functions), and cut-offs of 23/24 have typically been used to show significant cognitive impairment. It has been widely translated and used. A standardized version [42,43] improves its reliability, and is probably most important in research settings. Although it is not useful for diagnostic purposes, the MMSE is widely used in detecting change in clinical work and in research studies [44,45]. The TMT is a neuropsychological test composed of two parts: in part A (TMTA), subjects must connect 25 numbered circles, and in part B (TMTB), numbers and letters and the score is the time taken to complete each part. The Trail Making Test (TMT) provides information on processing speed, mental flexibility, and executive functions. The measurement of TMT part A was used to assess processing speed whereas the TMT-B provides a measurement of the executive function domain, including divided attention [23,42].

### 2.3. Evaluation of Neuropathy and Foot Alterations

We evaluated several foot alterations consisting of the most frequently observed in diabetic foot. The descriptions of these variables are presented in Table 1. The ambulatory screening of the diabetic neuropathy was diagnosed clinically by a trained physician and podiatrist according to the validated system of the Michigan Neuropathy Screening Instrument (MNSI), in which higher MNSI scores mean more severe neuropathy [43].

### 2.4. Statistical Analysis

The quantitative variables were subjected to a descriptive analysis using central tendency and dispersion measures (mean and standard deviation from the mean). Likewise, a descriptive analysis was also performed for the qualitative variables based on frequency distributions. The Kolmogorov–Smirnov test was used to estimate the normal distribution of quantitative variables and thus to define the type of test to be used (parametric or nonparametric). The differences between the means of two or more groups were analyzed using nonparametric tests (the Mann–Whitney U test). The correlation between quantitative variables was evaluated using the nonparametric Spearman’s correlation. A receiver operating characteristic curve (ROC curve) was used to assess the diagnostic ability of the neuropathy severity level (MNSI score) and glycemic control (Hb1Ac) as suitable variables for predicting adherence to self-care behaviors during the follow-up visit. The confidence level used for all the analyses was 95%, with a statistical significance of *p* < 0.05. The IBM SPSS statistical package (version 24.0) was used for all the statistical analyses.

## 3. Results

### 3.1. Characteristics of the Study Sample

The sociodemographic and clinical features of the diabetic patients are shown in Table 2.

The sample included adults and older individuals with diabetes. A total of 35 individuals were aged 65 or over (72.9% of the study sample), with different times elapsed since the diabetes diagnosis (91.7% of the sample suffered from type II diabetes). As regards the body-mass index (BMI), 33.3% (n = 16) had normal weight, 29.2% had overweight (n = 14), and 37.5% had obesity (n = 37.5%). The cut-off of ≤7% for HbA1C as a surrogate for acceptable control of diabetes [40] was demonstrated in 20 individuals (42.6%), whereas 27 individuals (57.4%) had HbA1C above the recommended level of 7%, indicating a poor glycemic control. For diabetic peripheral neuropathy (DPN), considering a cut-off of 7 score in the MNSI [46], 20 individuals (42.6%) had a relevant DPN. The prevalence of foot alterations is shown in Table 1. The most prevalent foot alterations were nail diseases and xerosis, followed by overload areas and hyperkeratosis. There were significant differences in age between the patients with or without nail disease (*p* = 0.031, Mann–Whitney test), with or without hyperkeratosis (*p* = 0.007, Mann–Whitney test), dry skin on the foot (*p* = 0.025, Mann–Whitney test), with or without overloaded plantar areas (*p* = 0.025, Mann–Whitney test), and with or without ulcerative lesions (*p* = 0.023, Mann–Whitney test). There were significant differences in the time elapsed (number of years) since the diabetes diagnosis between the patients with or without amputation (*p* = 0.021, Mann–Whitney test). No significant differences were observed for HbA1C and BMI between patients with and without foot alterations (*p* > 0.05 in all cases).

### 3.2. Cognitive Function

**Figure 1:**
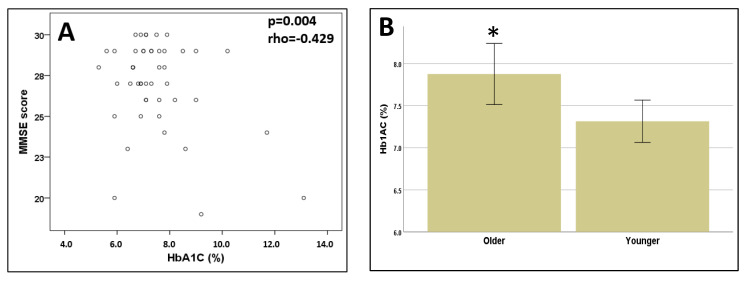
(**A**) Correlation between MMSE score and glycemic controls (expressed as Hb1Ac values). (**B**) Differences between Hb1Ac values in young (≤65 years old) and older (≥65 years old) patients. The asterisk “*” means significant difference *p* < 0.05. Hb1Ac: glycated haemoglobin; MMSE: mini mental state examination.

The mean score on the MMSE was 27.0 ± 0.4. Although it is not diagnostic, the cut-off score suggesting a diagnosis of neurocognitive disorders is established as a score of 23/24 points in persons over 65 years old, and 27/28 points in individuals under 65 adjusted for educational level [47,48]. Based on this assumption, we dichotomized the variable MMSE score into individuals with or without cognitive impairment, and we found N = 10 individuals (21.3%) fulfilled the criteria of cognitive impairment. The mean TMT-A score was 61.7 ± 3.1 s (range 20–130) and for the TMT-B it was 196.7 ± 15.2 s (range 45–300 s). A cutoff time of 300 s is generally used to discontinue test administration, and is therefore the typical maximum score, and the errors on the TMT do not directly contribute to the scoring and are generally not tallied [49,50]. However, some recent reports have identified cut-offs of TMT in older individuals in order to predict unsafe driving, where the mere inability to complete the test in a reasonable time frame (e.g., TMT-A > 48 s or TMT-B > 108 s) may still be a useful tool in separating unsafe from safe or marginal drivers in those samples [51]. By applying these cut-off points, 70.2% (N = 33) of the sample have an impairment of executive function/attention task in both the TMT-A and TMT-B tests. Taking age and education into account as confounders, there was a significant correlation between TMT-A and TMT-B with MMSE scores (*p* = 0.016, rho = −0.361) and *p* = 0.011, rho = −0.380), respectively). There were significant differences in TMT-B scores between male and female patients (*p* = 0.01, Mann–Whitney test). For the other cognitive tests (TMT-A and MMSE) there were no significant differences between sexes (*p* > 0.05) in all cases. No significant correlations were found between the TMT-A, TMT-B and HB1Ac (*p* = 0.567, rho = 0.089); *p* = 0.420, rho = 0.125). In contrast, a strong correlation was found between the MMSE and HB1Ac (*p* = 0.004, rho = −0.429) (Figure 1A).

BMI was not correlated to any cognitive test (TMT-A, TMT-B, and MMSE) (*p* > 0.05 in all cases). No significant differences were found in cognitive test scores based on the type of diabetes (TMT-A: *p* = 0.166; TMT-B: *p* = 0.605; MMSE: *p* = 0.886, Mann–Whitney test), or between patients aged ≥ 65 years and patients < 65 years (TMT-A: *p* = 0.232; TMT-B: *p* = 0.249; MMSE: *p* = 0.556, Mann–Whitney test).

### 3.3. Relationship between Foot Alterations and Cognitive Function

There was no significant correlation between the MNSI and TMT-A (*p* = 0.983, rho = −0.003), TMT-B (*p* = 0.350, rho = 0.139) or MMSE scores (*p* = 0.653, rho = 0.067), nor any differences in any variable (TMT-A, TMT-B, MMSE, age Hb1AC, BMI, years with diabetes diagnosis) based on categorization of the MNSI based on the cut-off score of 7 points (*p* > 0.05 in all cases).

The MNSI score was not significantly correlated with age (*p* = 0.109, rho = −0.237), Hb1AC (*p* = 0.716, rho = 0.055), BMI (*p* = 0.436 (rho = 0.116). However, when we categorized the age for patients aged ≥ 65 years and patients < 65 years, we found that older patients had a higher MNSI score and higher Hb1Ac values than younger patients (*p* = 0.01, Mann–Whitney test) (Figure 1B). No significant differences were observed for the MMSE, TMT-A, TMT-B, MNSI scores based on the presence or absence of any particular type of foot alteration (*p* > 0.05 in all cases).

### 3.4. Adherence to Treatment at the Follow-Up Visit

**Figure 2:**
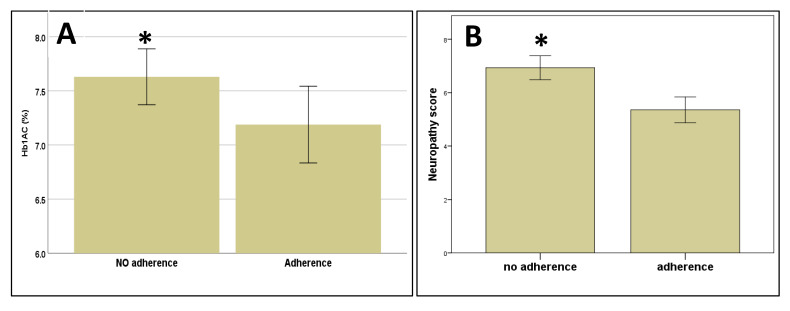
Differences in glycemic controls (expressed as Hb1Ac values) (**A**) and MNSI score (neuropathy score) (**B**) between patients with good adherence and no adherence to treatment and self-care behaviors for diabetic foot alterations. The asterisk “*” means significant difference *p* < 0.05. Hb1Ac: glycated haemoglobin; MNSI: Michigan neuropathy screening instrument.

During the follow-up visit, we evaluated the adherence to foot care treatment, and we evaluated whether clinical variables influence adherence or not. Patients with no adherence to the foot treatment have a significantly higher score for MNSI and HbA1C levels compared to patients with good adherence to the treatment (*p* = 0.027 and *p* = 0.039, Mann–Whitney test, respectively) (Figure 2A,B).

However, it is important to acknowledge that HbA1C reflects glucose control for the 3 months before the test.

**Figure 3:**
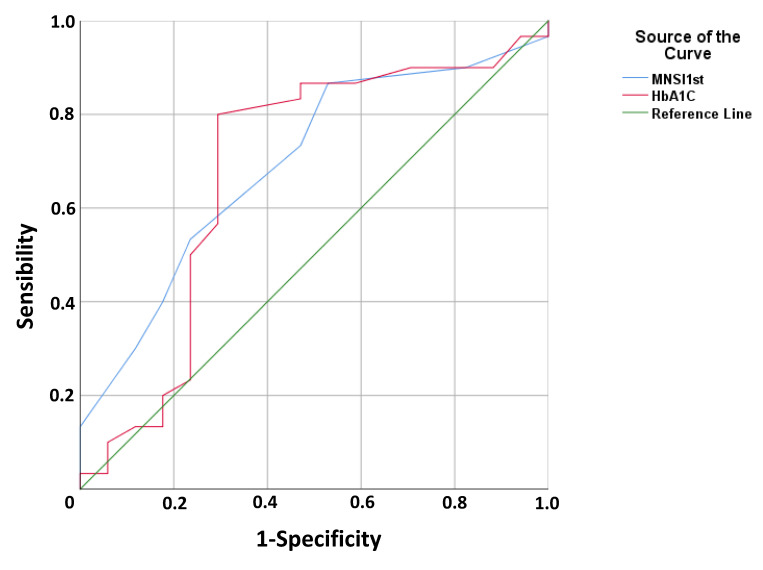
Receiving operating curve analysis for the sensitivity and specificity of the MNS score and Hb1Ac values to discriminate between patients with good and poor adherence to treatment for foot alterations. Hb1Ac: glycated haemoglobin; MNSI: Michigan neuropathy screening instrument.

In addition, when we analyzed adherence or otherwise and these variables were dichotomized using cut-off points (7 point for MNSI and HbA1C ≤ 7% for HbA1C as a surrogate of acceptable control of diabetes) we still observed significant differences (*p* = 0.003 and *p* = 0.04, Chi-squared test, respectively). No differences were observed between adherence and the scores in MMSE (*p* = 0.78), TMT-A (*p* = 0.85), TMT-B (*p* = 0.26) test, and likewise no significant differences were observed when analyzing cognitive function tests as a dichotomized variable based on their cut-off scores (*p* > 0.05 in all cases). No significant differences were found between adherence to treatment at follow-up visit and age (*p* = 0.39, Mann–Whitney test), educational level (*p* = 0.58, Chi-squared test), sex (*p* = 0.89, Chi-squared test), or type of diabetes (*p* = 0.18, Chi-squared test). No significant differences were found between adherence to treatment and specific foot alterations (*p* > 0.05 in all cases, Chi-squared tests). In order to identify the ability of neuropathy severity level (MNSI score) and glycemic control (Hb1Ac) as suitable variables for predicting adherence to self-care behaviors as a cornerstone for the treatment of foot alterations in patients with diabetes, we performed receiver operating characteristic (ROC) curve analysis (Figure 3). For the MNSI score, the value of the ± SEM area under the curve was 0.694 ± 0.079, with CI 95% 0.538–0.873 (Figure 3) with acceptable values, and the best cut-off value is 5.5 points with a sensitivity to discriminate adherence to the treatment of 73.3% and a specificity of 64.7%. For Hb1Ac, the value of the ±SEM area under the curve was 0.681 ± 0.090, with CI 95% 0.504–0.858 (Figure 3) and the best Hb1Ac cut-off value is 6.95% with a sensitivity of 80.0% and a specificity of 70.6%.

## 4. Discussion

Previous research has shown that diabetes may cause cognitive impairment [40,52] and accelerate the progression of cognitive impairment and/or dementia [41]. Diabetic foot is one of the most common complications, and foot ulcers and skin diseases are indicative of severe diabetes [53]. Few studies have shown an association between DPN, a decline in MMSE and TMT scores and cutaneous impairment [54,55], and the relationship between all these factors and adherence to the clinician’s recommendations for self-care behaviors in people with diabetic foot complication is quite new and has been investigated in diabetic patients who have undergone amputations or been hospitalized for diabetic foot ulcers [23,24]. Although the MMSE and TMT scores were correlated with each other, which confers the concurrent validity for measuring cognitive function in DPN patients on each scale, our results showed that a proper glycemic control measured as HB1Ac level < 7% [56] was only correlated with the MMSE score, suggesting that prefrontal cortex functions evaluated by TMT are less sensitive to the effect of sustained hyperglycemia. However, a more thorough analysis of executive functions in future studies should also take into account other neuropsychological tests related to this cognitive domain, such as the Verbal Fluency Test (VFT)—F, A and S; the Digits Forward and Backward subtests; the Stroop Test; the Wisconsin Card Sorting Test (WCST) among others [57]. Our results confirm recent findings in patients hospitalized for diabetic foot ulcers, showing the level of general cognitive abilities measured with another screening tool for cognitive impairment e.g., the Montreal Cognitive Assessment with improved knowledge of peripheral neuropathy and foot ulcers [23]. These differential effects on cognitive tests may reflect the effects of hyperglycemia in different areas of the brain as suggested by the fact the higher HbA1c was associated with poorer executive function (measured in TMT in our study) only among individuals with cognitive impairment [48]. In our study, the finding that the significant association between HbA1c and TMT in individuals who already have cognitive impairment (low MMSE scores) suggests that glycoregulation is related to cognitive performance predominantly after the onset of cognitive impairment. Both hyper- and hypoglycemia in diabetes patients seem to play different and overlapping roles in cognitive impairment, and the vulnerability of different brain areas to hyper-and hypoglycemia needs further studies [58,59] and other pathophysiological mechanisms can have different effects on diabetes-induced cognitive impairment which may be associated with or exacerbated by cardiovascular disease, including hypertension [60] and cerebral vascular complications [61]. Older age is associated with both a reduced cognitive ability and impairment in nerve function due to ageing process, and diabetes-deleterious alterations in the nervous system may exacerbate such physiological changes [62]. However, our results showed that cognitive function and neuropathy are not related in diabetic patients suggesting these two nervous system alterations may have different mechanisms that need to be further elucidated [55]. Although these complications do share some similarities, multiple and individual components may play an important role in determining who will develop central and who will develop peripheral nerve destruction.

Cutaneous alterations and DPN are a particular feature of diabetic foot complications. In this study, we found that elderly patients (aged ≥ 65) with foot disorders present higher levels of cognitive alteration compared to younger subjects, with the exception of HbAc1, which seems be related to the existence of diabetic foot disorders and the aging process [63,64,65]. However, a more thorough evaluation of cognitive functions will be required for analysis of cognitive functions, involving several neuropsychological tests and their influence on self-care in patients with diabetic foot.

According to the literature, cognitive alterations have shown to increase diabetic foot complications and inadequate self-care; these studies are consistent with the results obtained [11,54,55]. In this study, we found a prevalence of some cutaneous impairments such as nail disease, xerosis, ulcerative lesion, hyperkeratosis, and overloaded plantar areas in elderly patients. We also confirmed that the duration (number of years) of diabetes is associated with amputation in the lower extremities [66]. Overweight/obesity was not associated with any outcomes in patients with or without foot alterations according to the literature, and glycated hemoglobin levels are not negatively influenced by BMI in diabetic patients [67].

In this study, poorly controlled glycemic levels (high Hb1Ac > 7%) and higher DPN scores predicted adherence to self-care behaviors in the follow-up visit, and although cognitive impairment is a crucial predictor of adherence to medical education programs, the severity of cognitive dysfunction in patients with diabetic foot cannot fully account for a reduced adherence, and other social factors and mood disorders may affect these outcomes [12,13,14].

The American Diabetes Association has suggested using HbA1C values to determine glucose control, treatment, and diet [68,69,70]. Our study identified an Hb1Ac level of approximately 7% and a neuropathy score of about 5.5 in the MNSI as the best-cut off points to discriminate poor adherence to treatment for foot complications, and future longitudinal studies with larger follow-up should validate these cut-off values as suitable for diagnosis of foot complications and their evolution over time. Sodi et al. [71] have recently pinpointed the potential limitations of the use of HbA1c in monitoring glycemic control over time, and further studies should use other measures of average glycemia, such as plasma fructosamine or glycated albumin which are quite sensitive and useful alternatives for estimating glycemic control in diabetic patients and related research. Despite the limitations of our study, such as the limited sample size, neuropathy and foot alterations seem to be unrelated to cognitive dysfunction and overweight/obesity, and provide some important guidelines for the future in this research field. The relationship between foot alterations and other concomitant micro- and macrovascular complications such as a nephropathy or retinopathy [72,73] will require future research. Finally, the role of depression, which is common in type II diabetes patients [12,13,14], requires a more in-depth analysis of the role of cognitive dysfunction and foot alterations, since it has been associated with higher scores for frailty [74,75,76] and may in turn be associated with poor adherence to treatment for foot complications.

## 5. Conclusions

This study showed that lower measures of cognitive functions with a generalized measure of basic cognitive proficiency and psychomotor processing speed, executive function and scanning task are associated with cutaneous alterations and foot disorders in older (65 years and over) diabetic patients. Poorly controlled glycemic levels (high Hb1Ac > 7%) and higher neuropathy (DPN) scores predicted the degree of adherence to self-care behaviors, podiatric educational programs, and daily foot care management. Strategies to increase adherence among patients with diabetic foot should be individualized, while also considering glycemic levels and DPN scores. Our findings concerning cutaneous alterations suggest that healthcare professionals should pay greater attention to cognitive impairment. This study supports the need to provide a diabetes teaching program and follow-up considering the evaluation of cognitive functions during diabetic foot screening and risk assessment. High-quality studies are needed with a large population to evaluate interventions to increase therapeutic adherence among people with diabetic foot complications.

## Figures and Tables

**Table 1:** The descriptions of cutaneous impairment variables.

Cutaneous Impairment and Appearance of Feet According to the Michigan Neuropathy Screening Instrument	
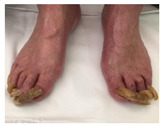	**Nail diseases**: diseases of the nail plate and tissues surrounding it.
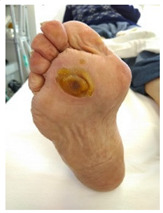	**Hyperkeratosis**: hyperkeratosis is caused by excessive mechanical loading. The hyperkeratosis (or callus) is thickened skin leading to a further increase in the loading of the foot, often with subcutaneous hemorrhage and eventually skin ulceration.
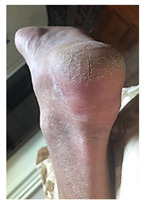	**Skin care and dryness (xerosis)**: abnormal dryness of foot tissues caused by a lack of moisture in the skin (which can be treated by emollient therapy).
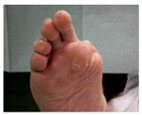	**Amputation**: resection of a segment of a limb through a bone or through a joint.
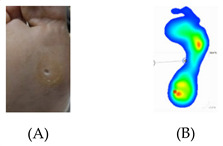	**Overload areas**: (A) a mechanical stress in some areas, the response to which is usually represented by a thickened skin callus; (B) peak pressures evaluated with baropodometric platform.
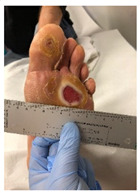	**Foot ulceration**: a break in the skin of the foot that involves at least the epidermis and part of the dermis. We considered the foot ulceration in people with currently or previously diagnosed diabetes mellitus.
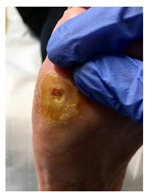	**Pre ulcerative lesions**: a foot lesion with a high risk of developing into a foot ulcer, such as intra- or subcutaneous hemorrhage, blister, or skin fissure not penetrating the dermis.

**Table 2:** Sociodemographic and clinical features of diabetic patients.

**Age**	Mean ± SD: 70.8 ± 1.7 years (Minimum 42–Maximum 88)
**Sex**	27 male; 27 female
**Educational level: number of individuals.**	Primary school: 40 Secondary school: 6 University: 1
**Type of Diabetes**	Type I: 3 individuals Type II: 44 individuals
**Years diagnosed with diabetes**	Mean ± SD: 18.8 ± 1.7 years (Minimum 2–Maximum 47)
**Glycated hemoglobin (HbA1C; %)**	Mean ± SE: 7.5% ± 0.2% (Minimum 5.3–Maximum 13.1)
**BMI**	Mean ± SD: 27.8 ± 0.7 (Minimum 20.0–Maximum 37.9)
**Type of foot alterations**	Nail diseases: 31 of 47 individuals (64.6%) Hyperkeratosis: 22 of 47 individuals (45.8%) Skin care and dryness (xerosis): 37 of 47 individuals (77.1%) Amputation: 10 of 47 individuals (20.8%) Overload areas: 23 of 47 individuals (47.9%) Foot ulceration: 5 of 47 individuals (10.4%) Pre-ulcerative lesions: 3 of 47 individuals (6.3%)
**Neuropathy score**	Mean ± SD: 6.4 ± 0.4 (Minimum 2–Maximum 13)
